# Enhanced High-Performance iPP/TPU/MWCNT Nanocomposite for Electromagnetic Interference Shielding

**DOI:** 10.3390/polym16131837

**Published:** 2024-06-27

**Authors:** Yanru Li, Wenting Yu, Qian Ruan, Kun Li, Xiaoqin Guo, Zhongyi Bai, Jingbo Chen

**Affiliations:** 1 School of Materials Science and Engineering, Zhengzhou University, Zhengzhou 450001, China; 18240508329@163.com (Y.L.); yuwentingzzu@163.com (W.Y.); rq132753@gs.zzu.edu.cn (Q.R.); chenjb@zzu.edu.cn (J.C.); 2 School of Mechatronics Engineering, Zhengzhou University of Aeronautics, Zhengzhou 450046, China; guoxq@zua.edu.cn (X.G.); zhybai@zua.edu.cn (Z.B.)

**Keywords:** melt blending, polypropylene-based nanocomposites, EMI shielding property, iPP/TPU/MWCNTs, bicontinuous structure

## Abstract

The rapid development of electronic communication technology has led to an undeniable issue of electromagnetic pollution, prompting widespread attention from researchers to the study of electromagnetic shielding materials. Herein, a simple and feasible method of melt blending was applied to prepare iPP/TPU/MWCNT nanocomposites with excellent electromagnetic shielding performance. The addition of maleic anhydride-grafted polypropylene (PP-g-MAH) effectively improved the interface compatibility of iPP and TPU. A double continuous structure within the matrix was achieved by controlling the iPP/TPU ratio at 4:6, while the incorporation of multi-walled carbon nanotubes endowed the composites with improved electromagnetic shielding properties. Furthermore, by regulating the addition sequence of raw materials during the melt-blending process, a selective distribution of carbon nanotubes in the TPU matrix was achieved, thereby constructing interconnected conductive networks within the composites, significantly enhancing the electromagnetic shielding performance of iPP/TPU/MWCNTs, which achieved a maximum EMI shielding efficiency of 37.8 dB at an iPP/TPU ratio of 4:6 and an MWCNT concentration of 10 wt.%.

## 1. Introduction

In recent decades, the rapid development of information and communication technology (ICT) and electronic devices has brought great convenience to human daily life and industrial production. Nevertheless, the broad application of such apparatuses also engenders concerns regarding the deleterious impact of the associated electromagnetic waves on human beings and the optimal operation of sensitive electronic systems [1,2]. Prolonged exposure of the human organism to electromagnetic wave environments predisposes it to DNA mutagenesis [3], thereby potentially instigating a spectrum of illness [4]. Consequently, the study of materials endowed with outstanding electromagnetic shielding properties, capable of safeguarding human health and the normal work of precision devices, is of significant importance. Metal materials usually exhibit excellent electromagnetic shielding performance due to their high conductivity [5]. However, the numerous drawbacks, including high density, difficult molding, harsh processing conditions, poor corrosion resistance, and high cost, are severely restricting their development and application in a broader range of fields [6]. In comparison, polymer materials possess numerous advantages over metals, such as low cost, light weight, easy processing conditions, good corrosion resistance, and so on [7,8], and thus have garnered widespread applications across diverse domains of human daily life and industrial operations. However, the intrinsic insulativity of most polymer materials causes them to have very low electrical conductivity, which has restricted the possible application of most polymers in the field of EMI shielding. Thus, the preparation of conductive polymer nanocomposites by adding various conductive and magnetic nanoparticles, such as carbon nanotubes (CNTs) [9,10,11,12], graphene nanosheets (GNSs) [13,14], silver nanoparticles [5,15], MXenes [16,17,18], etc., is currently a research hotspot in the field of EMI shielding materials.

Carbon-based nanomaterials, due to their numerous advantages, are widely employed in the preparation of electromagnetic shielding polymer composite materials [19,20]. Among these, carbon nanotubes, characterized by mature fabrication techniques, relatively low production costs, and high electrical conductivity, stand out as one of the most commonly used conductive materials in current production of EMI polymer composites. CNTs can be categorized into single-walled carbon nanotubes (SWCNTs) and multi-walled carbon nanotubes (MWCNTs) according to the number of concentric tubular layers [21,22]. Comparative analysis has revealed that SWCNTs exhibit a more uniform diameter distribution with diminished defects, while MWCNTs boast superior length-to-diameter ratios, with lengths extending several centimeters, and are less prone to agglomeration [23,24,25]. As MWCNTs are more affordable, they are more commonly used in experimental investigations.

Nevertheless, the substantial surface energy inherent in carbon nanotubes frequently engenders poor dispersion within polymer matrices, thus engendering interfacial resistance between carbon nanotubes and resin matrices, which impedes electron transfer and culminates in diminished conductivity of carbon-nanotube-infused polymer matrix composites relative to expectations [26]. Concurrently, elevated concentrations of carbon nanotubes (CNTs) within the polymer matrix precipitate aggregation, engendering a scenario wherein composite material performance deteriorates with escalating CNT concentration, which has significantly constrained the utilization of CNTs in the field of high-performance polymer nanocomposite fabrication [23,27]. Consequently, the uniform dispersion of high-concentration carbon nanotubes within polymer matrices represents a pivotal challenge currently being confronted.

A prerequisite for two polymers to be able to form a bicontinuous structure is that they are poorly compatible or incompatible. In the case of isotactic polypropylene (iPP), the methyl groups along the molecular chain are consistently positioned on one side, resulting in a high degree of stereoregularity. This structural regularity, coupled with the corresponding high degree of crystallinity, contributes to the elevated melting point of iPP, which can reach up to 167 °C. Due to its excellent processing stability, favorable comprehensive mechanical properties, high deformation temperature, and relatively low cost, iPP is extensively utilized in the industry as a commercial thermoplastic material [28]. Conversely, thermoplastic polyurethane (TPU) is an elastomeric material that bridges the properties of conventional rubber and plastics. It is characterized by high strength, high hardness, a high modulus, and significant elongation [29]. Given the substantial differences in the properties of iPP and TPU, their inherent incompatibility, and their closely aligned processing temperatures, these two polymers fulfill the prerequisites for forming a bicontinuous phase.

Shi et al. [30] conducted a comparative study on the dispersion of MWCNTs in poly(L-lactic acid) (PLLA) and iPP matrices. Their findings indicated that MWCNTs were better dispersed in PLLA matrices, and the percolation threshold of PLLA/MWCNT nanocomposites was significantly lower than that of iPP/MWCNT nanocomposites. Moreover, the EMI shielding performance was superior in PLLA matrices. Zhang et al. [31] investigated an eco-friendly method involving pre-coating, melt mixing, and injection molding. They discovered that a segregated CNT/PP composite containing only 3.5 wt.% CNTs exhibited an average EMI shielding effectiveness (EMI SE) of 32 dB. This performance marked a 130% improvement compared to the 14 dB achieved by CNT/PP composites prepared via conventional injection molding and a 30% improvement over the 25 dB obtained through compression molding. Beomsu et al. [32] provided a comprehensive evaluation of various CNT-based TPU composites aimed at enhancing EMI SE and thermal management. They reported that a composite with long CNTs (10 wt.%) demonstrated an impressive EMI shielding efficiency of 42.5 dB. Conversely, a composite with short CNTs exhibited a thermal conductivity of 0.51 W/mK, with the corresponding thermal conductivity enhancement efficiency exceeding 145% relative to pure TPU. Wang et al. [33] developed multilayer TPU/MWCNT composite foams with a gradient structure for EMI shielding applications. The average EMI SE of the TPU/MWCNT composites with a gradient structure was 1.2 times greater than that of homogeneous composites. Furthermore, after foaming, the average EMI SE of the gradient foams surpassed that of the homogeneous foams, reaching a maximum average EMI SE of 35.4 dB.

Due to the significant disparity in compatibility between iPP and TPU, the overall performance of their composites is often adversely affected. In this study, maleic anhydride-grafted polypropylene (PP-g-MAH) was employed as a two-phase compatibilizer to enhance the properties of the composites. PP-g-MAH was synthesized via reactive extrusion, where polypropylene was grafted with maleic anhydride, introducing strong polar side groups onto the non-polar polypropylene backbone. This modification enables PP-g-MAH to act as an intermediary, improving adhesion and compatibility between polar and non-polar materials [34,35]. Incorporating PP-g-MAH in polypropylene production significantly enhances the filler–polypropylene affinity and filler dispersion. Consequently, this improves the dispersion of fillers within polypropylene, thereby increasing the tensile and impact strength of the filled polypropylene. In this experiment, iPP was used as one of the matrices, and the addition of PP-g-MAH as a compatibilizer was expected to substantially improve the compatibility between iPP and TPU.

In this work, iPP and TPU were used as the matrix, and MWCNTs were selected as conductive fillers. Based on the different dispersions of MWCNTs in iPP and TPU, the TPU/MWCNT composites were first prepared as the new filler to be added into iPP matrix. Moreover, the effect of the compatibilizer PP-g-MAH, on the compatibility of iPP and TPU was explored, as were the final EMI shielding properties.

## 2. Materials and Methods

### 2.1. Materials

Isotactic polypropylene (iPP, brand T30S) with a melting temperature of around 163 °C and a density range of 0.85~0.92 g/cm^3^, produced by Ningxia Petrochemical (Ningxia, China), was used. Thermoplastic polyurethane (TPU, brand 8792a) with a softening point of about 100 °C and a density of 1.20 g/cm^3^, produced by BASF (Ludwigshafen, Germany), was purchased. Multi-walled carbon nanotubes (MWCNTs, brand TNM2) with a density of 2.1 g/cm^3^ and purity > 95% were produced by Chengdu Organic Chemical Co. Ltd. (Chengdu, China). Maleic anhydride-grafted polypropylene (PP-g-MAH, brand CMG9801) with a density of 0.90 g/cm^3^ and a grafting rate of 0.5–1.0 wt.% was supplied by Shanghai Jiayirong Polymer Co, Ltd. (Shanghai, China). 

### 2.2. Preparation of the Composites

#### 2.2.1. Preparation of iPP/MWCNT, TPU/MWCNT, and iPP/TPU Composites

iPP/MWCNT, TPU/MWCNT, and iPP/TPU composites with different ratios were first prepared by a simple melt-blending process at 190 °C in a torque rheometer (CRT-100, Shanghai Sierda Scientific Instrument Co., Shanghai, China) with a rotating speed of 50 r/min for 12 min to blend them well. Standard stretch specimen of different composite materials, with a thickness of 2.5 mm, were prepared by using a plate vulcanizer (XLB-D Qingdao Xinben Technology Co., Qingdao, China) at a temperature of 200 °C under 10 MPa pressure for 8 min.

#### 2.2.2. Preparation of iPP/TPU/MWCNT Nanocomposites

**Figure 1:**
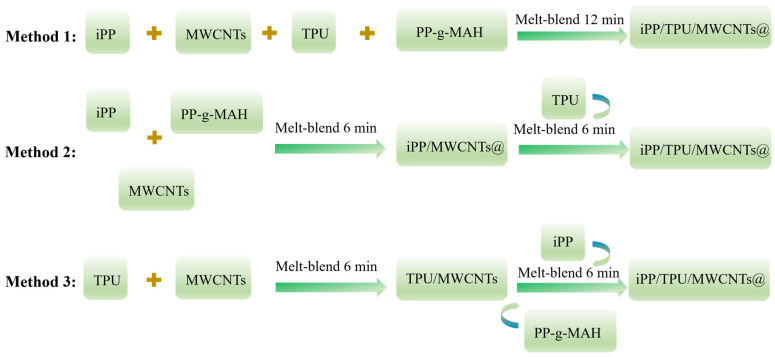
Schematic diagram of the preparation processes for three different compounding methods.

iPP/TPU/MWCNT composites were prepared by sequential blending method, for which three distinct melt-blending techniques were devised, as depicted in Figure 1:

Method 1: Concurrently add iPP, TPU, PP-g-MAH, and MWCNTs to the torque rheometer and melt-blend for 12 min.

Method 2: Initially, add iPP, PP-g-MAH, and MWCNTs to the torque rheometer and melt-blend for 6 min. Subsequently, introduce TPU and continue co-mixing for an additional 6 min.

Method 3: First, add TPU and MWCNTs to the torque rheometer and melt-blend for 6 min. Then, add iPP and PP-g-MAH and proceed with melt blending for another 6 min.

#### 2.2.3. Preparation of iPP/TPU/MWCNT/PP-g-MAH Composites

The iPP/TPU/MWCNT/PP-g-MAH composites were also prepared by a sequential blending method, in which TPU and MWCNTs were melt-blended in the first 6 min, after which iPP and PP-g-MAH were added to continue the mixing process for another 6 min. The subsequent steps were the same as presented in Section 2.2.2.

### 2.3. Density Test

The density of the sample was tested using the drainage method as follows Equation (1):

(1) $$\rho =\frac{m_{a}}{m_{a}-m_{b}}\rho_{0}$$

where *ρ* is the density of the sample (g/cm^3^), *ρ*_0_ is the density of water (g/cm^3^), *m_a_* is the mass of the sample in air, and *m_b_* is the mass of the sample in water.

### 2.4. Volumetric Conductivity Test

The digital multimeter (DMM4050, Tektronix, Beaverton, OR, USA) was used to test the resistance *R* (Ω) of the samples by applying a small amount of conductive silver adhesive to the contact between the sample and the test probe to eliminate the contact resistance between the test probe and the sample.

A small amount of conductive silver gel was applied to the contact area between the sample and the test probe to eliminate the contact resistance between the test probe and the sample. The volumetric conductivity *σ* (S/cm) was calculated by Equation (2); *L* is the length of the sample (cm), and *S* is the cross-sectional area of the sample (cm^2^).

(2) $$\sigma =\frac{L}{R\cdot s}$$

The weight fraction of MWCNTs can be converted to volume fraction by Equation (3). *A* is the mass fraction of the sample (wt.%), *B* is the volume fraction of the sample (vol.%), and *ρ*_1_ is the density of the sample, while *ρ*_2_ is the density of MWCNTs, which is a constant value of 2.1 g/cm^3^.

(3) $$A=\frac{\rho_{1}\cdot B}{\rho_{2}}$$

### 2.5. Morphological Characterization

The morphologies of the specimens were observed and recorded with a scanning electron microscope (SEM, FEI Quatan 200, FEI Company, Hillsboro, OR, USA). The prepared samples were cut to the appropriate size and immersed in liquid nitrogen for 1 h. The samples were then removed and quenched quickly to preserve the complete microscopic morphology of the samples. Fracture surfaces were sputtered with gold to provide enhanced conductivity prior to SEM observation.

### 2.6. EMI Shielding Performance

The EMI shielding performance of the composites was measured in the frequency range of 18–26.5 GHz (K-band) at room temperature, using a vector network analyzer (VNA, Agilent N5234A, Santa Clara, CA, USA). Samples were cut into a size of 10.6 mm (Length) × 4.3 mm (Width) × *d* (Thickness); *d* (mm) varied according to requirements. Based on the Schelkunoff theory [36], the shielding effect of EMI shielding materials on EM waves is mainly based on three aspects, namely, reflection loss (*SE_R_*), absorption loss (*SE_A_*), and multiple reflection loss (*SE_M_*) [1,37]. The sum of *SE_A_*, *SE_R_*, and *SE_M_* is the total EMI SE (*SE_T_*). Notably, *SE_M_* usually can be ignored if the value of *SE_T_* exceeds 15 dB [38]. The total EMI SE is the sum of these three items. The EMI performance parameters, including *SE_T_*, *SE_R_*, and *SE_A_*_,_ were calculated from the scattering parameters *S*_11_ and *S*_21_, which are measurable quantities. According to Simon’s formula [39], the *SE_T_* can be written as Formulation (9), where *σ*, *f*, and *d* were the electrical conductivity, frequency, and thickness of the shielding materials, respectively. This equation shows that *SE_T_* is positively correlated with conductivity.

(4) $$R={\left|S_{11}\right|}^{2}$$

(5) $$T={\left|S_{21}\right|}^{2}$$

(6) $$SE_{R}=10\log\left(1-R\right)$$

(7) $$SE_{A}=10\log\left(\frac{T}{1-R}\right)$$

(8) $$SE_{T}=SE_{R}+SE_{A}$$

(9) $$SE_{T}=50+10\log\left(\frac{\delta }{f}\right)+1.7d\surd f$$

(10) R+A+T=1

## 3. Results and Discussion

### 3.1. Characterization of iPP/MWCNT and TPU/MWCNT Composites

#### 3.1.1. The Morphology of iPP/MWCNT and TPU/MWCNT Nanocomposites

**Figure 2:**
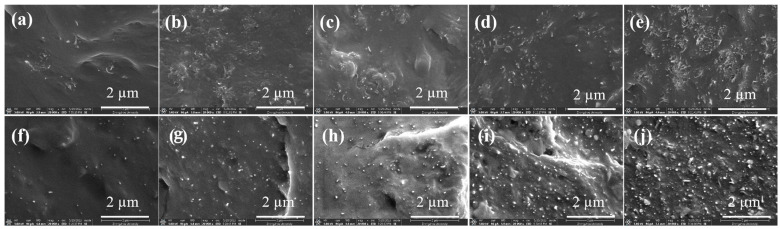
SEM images of iPP/MWCNT composites (**a**–**e**) and TPU/MWCNT composites (**f**–**j**), corresponding to MWCNT content of 2 wt.% (**a**,**f**), 4 wt.% (**b**,**g**), 6 wt.% (**c**,**h**), 8 wt.% (**d**,**i**), and 10 wt.% (**e**,**j**), respectively.

The dispersion and distribution state of conductive fillers in the polymer matrix had a crucial effect on the final properties of the composites. The cross-sectional morphology of iPP/CNTs and TPU/CNTs composites at different CNTs contents were shown in Figure 1. The agglomeration of MWCNTs could be clearly observed in iPP matrix with the increase content of MWCNTs from 2 wt.% to 10 wt.%, as shown in Figure 2a–e, while the largest agglomerates of MWCNTs were found in the iPP/MWCNT nanocomposite with MWCNTs of 10 wt.%, which reached about 1.0 μm (Figure 2e). In contrast, the dispersion of MWCNTs into the TPU matrix was apparently better than that of iPP. MWCNTs were found to uniformly dispersed into TPU matrix without significant agglomeration even for the TPU/MWCNT nanocomposite with MWCNT content of 10 wt.%. This large difference in MWCNT distribution into iPP and TPU matrix can be easily understood by considering their melt strength during the melt-blending process [27,28,40,41]. The high regularity and weak polarity of iPP molecular chains resulted in low melt strength, which led to weak shear force on MWCNTs during the melt blending process, ultimately leading to poor dispersion of MWCNTs within iPP matrix. While, in the cases of TPU/CNTs, due to lower molecular chain regularity and larger chain polarity of TPU compared to iPP, TPU chains can offer stronger shear forces on MWCNTs during the melt blending process, thus facilitating the better dispersion of MWCNTs within the TPU matrix, as shown in Figure 2j. 

#### 3.1.2. The Electrical and EMI Shielding Properties of iPP/MWCNT and TPU/MWCNT Nanocomposites

**Figure 3:**
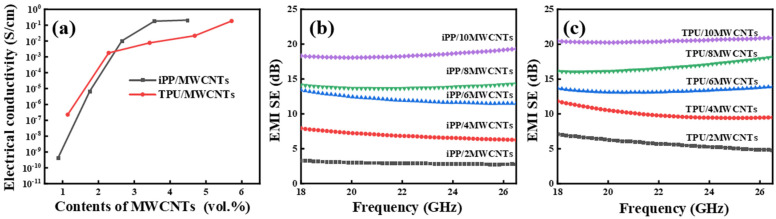
Variation of volume conductivity (**a**) and EMI shielding effectiveness (**b**,**c**) of conductive iPP/MWCNT and TPU/MWCNT composites with different MWCNT contents with the same sample thickness of 1.4 mm.

The electrical conductivity and electromagnetic shielding effectiveness (EMI SE) are two important and interrelated properties of electromagnetic shielding materials, typically associated with the content of conductive fillers and their dispersion state within the polymer matrix. We measured and calculated the electrical conductivity and electromagnetic shielding effectiveness of various polymer nanocomposites containing different contents of MWCNTs, the results were shown in Figure 3. As shown in the figures, the volumetric conductivity of both nanocomposites increased with increasing content of MWCNTs, reaching the percolation thresholds at about 3.0 vol.% for iPP/MWCNT and ca. 2.8 vol.% (Figure 3a) for TPU/MWCNT nanocomposites, proving that MWCNTs have a better connection network within TPU than in iPP matrix, which is consistent with the previous results from SEM images.

EMI shielding effectiveness is positively correlated with electrical conductivity, as shown in Figure 3b,c, the average total EMI shielding effectiveness (EMI *SE_T_*) of the composites also gradually increased with the increase of MWCNT content, reaching a maximum EMI *SE_T_* of approximately 19 dB for iPP/MWCNTs and approximately 21 dB for TPU/MWCNTs with both having an MWCNT content of 10 wt.%.

### 3.2. The Construction of Bicontinuous Phase Structure of iPP/TPU Blends

In biphasic immiscible polymer blend systems, there are usually two types of structures. One is the formation of the “sea-island” [42] structure when one component’s content is much lower than the other, and the other one is the bicontinuous phase structure when the content of both polymer components is comparable. During the preparation of polymer nanocomposites, the effective concentration of nanoparticles in the polymer nanocomposite can be effectively and significantly increased by controlling the distribution of nanofillers in single phase of the bicontinuous system, thereby enhancing the functional properties of the composite materials.

**Figure 4:**
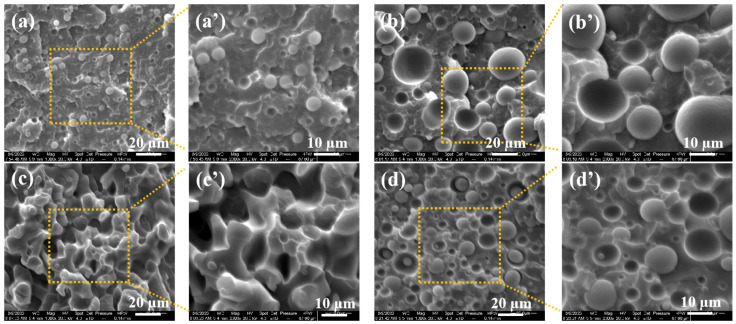
SEM images of iPP/TPU composites with iPP: TPU ratios of 8:2, 6:4, 4:6, and 2:8; (**a**–**d**) overall morphologies; (**a′**–**d′**) magnified images.

Furthermore, we prepared iPP/TPU blends with different iPP/TPU ratios from 8:2 to 2:8, to study the structural evolution; the results are shown in Figure 4. We can clearly see that when the iPP/TPU was 8:2, TPU acted as the dispersed phase due to its relatively low content, exhibiting a spherical dispersion phase within the iPP matrix (Figure 4a,a′). When the iPP/TPU ratio changed to 6:4, TPU remained insufficient to establish a continuous structure, maintaining spherical dispersion phase within the iPP matrix with a larger diameter of the “island” due to the increased content of TPU (Figure 4b,b′). With further escalation of TPU content, particularly at a 4:6 ratio, owing to density discrepancy between the two polymers, the volume fractions of both phases in the composite system became comparable, resulting in the formation of the bicontinuous phase structure, as illustrated in Figure 4c,c′. However, as TPU content continues to rise, a concomitant reduction in iPP content ensues. In this case, iPP served as the dispersed phase within the TPU matrix, thereby reinstating a “sea-island” structure (Figure 4d,d′).

#### Content of Compatibilizer

**Figure 5:**
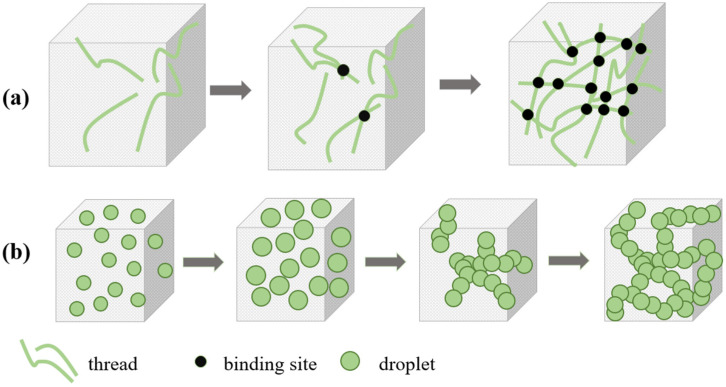
Schematic diagram of the formation of double-percolated structures by two immiscible phases. (**a**) Coalescence of threads; (**b**) coalescence of droplets.

The significant polarity discrepancy between iPP and TPU typically results in their incompatibility. For melts with high interfacial tension, during the stretching process, if the diameter is reduced to a certain extent, the surface tension becomes sufficient to cause the melt to break into small beads. In contrast, in two-phase blends with low interfacial tension, the formation of a bicontinuous phase occurs primarily through the fusion of microfibers. This is because the polymer melt phase is not easily severed during stretching, as illustrated in Figure 5a. Conversely, in blending systems with high interfacial tension, the microfibrous melt is unstable and readily transforms into spherical beads. Therefore, the bicontinuous phase in such systems is predominantly formed through the fusion of these spherical particles, as shown in Figure 5b [43]. Simple melt blending tends to limit the performance characteristics of the resulting composites. To address this issue, we employed PP-g-MAH as a compatibilizer, aiming to reduce the dispersed phase size and promote the formation of a double continuous structure [44]. Nonetheless, with increasing amounts of PP-g-MAH, the interfacial boundary between the two phases diminished progressively until it eventually disappeared. Consequently, it was imperative to determine the optimal concentration of PP-g-MAH necessary for the development of a stable bicontinuous phase structure.

**Figure 6:**
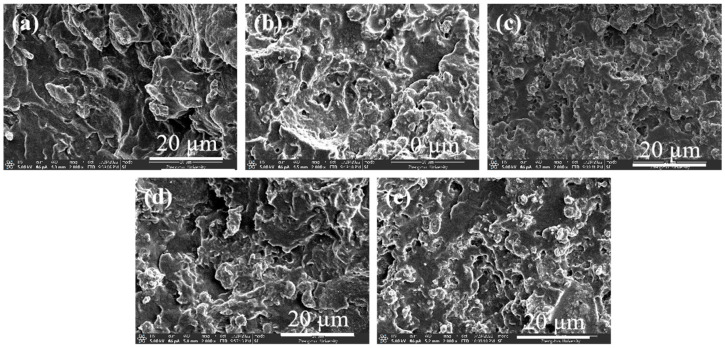
SEM images of iPP/TPU/MWCNT composites with iPP: TPU ratio of 4:6, MWCNT content of 10 wt.%, and different amounts of compatibilizer ((**a**–**e**) 0, 0.5, 1, 2, and 5 wt.%).

The samples incorporating the compatibilizer PP-g-MAH were designated as iPP/TPU/MWCNTs@. Figure 6a–e presents the cross-sectional morphology of iPP/TPU/MWCNTs@ with varying PP-g-MAH contents. As anticipated, an increase in PP-g-MAH content resulted in a reduction of the dispersed phase size and a gradual disappearance of phase boundaries. At a concentration of 5 wt.% (Figure 6e), the interface between iPP and TPU in certain regions had completely coalesced. This observation aligns with the regional cohesion theory previously discussed.

**Figure 7:**
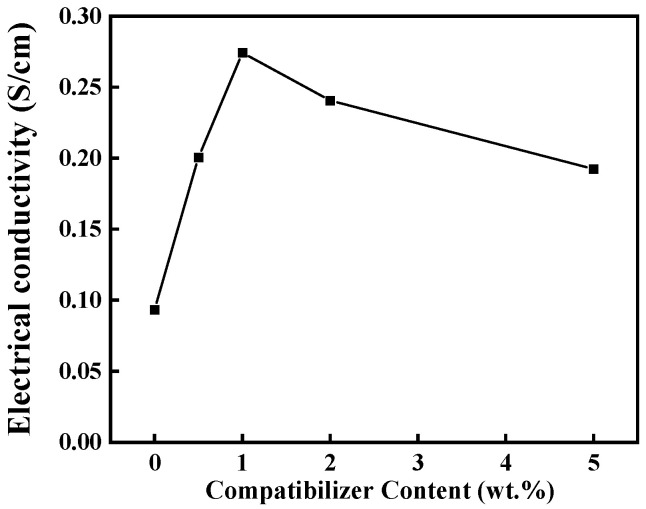
Volume conductivity of iPP/TPU/MWCNT composites with MWCNT content of 10 wt.% and different amounts of compatibilizer.

Figure 7 illustrates the effect of PP-g-MAH content on the volumetric conductivity of iPP/TPU/MWCNTs@. The highest volumetric conductivity of iPP/TPU/MWCNTs@ was observed at a PP-g-MAH content of 1 wt.%, exhibiting a value 2.8 times greater than that of iPP/TPU/MWCNTs. However, as the PP-g-MAH content increased to 5 wt.%, the volumetric conductivity decreased to 0.19 S/cm. It means that the optimal PP-g-MAH content was 1 wt.%. Correspondingly, the formation of the bicontinuous phase structure facilitated the establishment of conductive networks as well as the transfer of electrons. This finding underscores that the construction of a bicontinuous phase structure maximizes the efficacy of the conductive filler. Consequently, in subsequent studies, the PP-g-MAH content was fixed at 1 wt.%.

### 3.3. Effect of Processing Methods

Building on previous experimental findings, we further investigated the influence of various processing methods on the development of the bicontinuous phase structure, aiming to enhance the performance of iPP/TPU/MWCNT composites. The preparation process is shown in Section 2.2.2.

**Figure 8:**
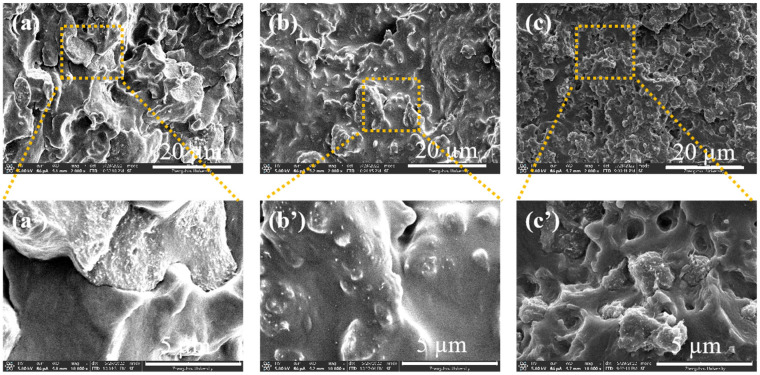
SEM images of the composites prepared by (**a**–**c**) methods 1, 2, 3; (**a′**–**c′**) are the magnified images.

To simplify the description, we designated these three processing conditions as Method 1, Method 2, and Method 3, respectively. The component content remained consistent across all three methods: the mass ratio of iPP to TPU was 4:6, MWCNTs constituted 10 wt.%, and PP-g-MAH content was 1 wt.%. For the samples obtained using Method 1 (Figure 8a,a′), it is challenging to clearly identify the phase in which the MWCNTs are distributed. Conversely, for samples prepared using both Method 2 (Figure 8b,b′) and Method 3 (Figure 8c,c′), the MWCNTs were predominantly distributed in the TPU phase. Additionally, the blends prepared using Method 3 exhibited finer particle structures.

This phenomenon can be elucidated by considering the previously mentioned melt strength. It is well established that TPU exhibits higher melt strength than iPP under identical processing conditions. In this context, melt strength can be understood as the capacity to “capture” MWCNTs. For the sample prepared using Method 1, since all components were added simultaneously, both TPU and iPP had the opportunity to capture portions of the MWCNTs. In contrast, samples prepared using Methods 2 and 3 involved two stages, resulting in different contact times and probabilities among the components. During the initial 6 min of Method 2, iPP captured a substantial portion, if not all, of the MWCNTs. However, due to the higher melt strength of the TPU phase and therefore its stronger capture ability, the TPU phase, despite being added later, managed to reallocate a portion of the MWCNTs already dispersed in the iPP phase. In other words, MWCNTs migrated from the iPP phase to the TPU phase. Nevertheless, not all MWCNTs migrated to the TPU phase, some remained within the iPP phase.

In Method 3, the MWCNTs were uniformly dispersed in the TPU during the initial processing stage. When the iPP phase was added later, the lower melt strength and capture ability of iPP did not facilitate the migration of MWCNTs. Consequently, in Method 3, the pre-mixing of TPU and MWCNTs resulted in an inseparable blend. During the subsequent melt-blending process with iPP, the TPU/MWCNT particles functioned as fillers.

**Figure 9:**
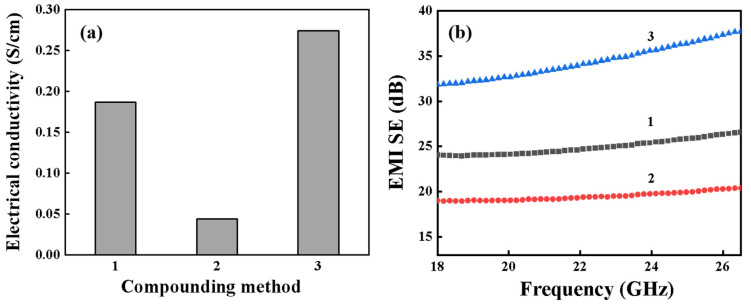
Volumetric conductivity (**a**) and electromagnetic shielding properties (**b**) of iPP/TPU/MWCNTs processed by three different methods with a sample thickness of 2.5 mm.

Figure 9 illustrates the volumetric conductivity and total electromagnetic shielding effectiveness (*SE_T_*) of samples produced via three distinct processing methods. The composites fabricated using Method 3 exhibited a volumetric conductivity of 0.27 S/cm, which is 6.7 times greater than that achieved by Method 1. Additionally, the average *SE_T_* value for samples with a thickness of 2.5 mm prepared by Method 3 reached 34.4 dB. In comparison, the average *SE_T_* values for samples prepared using Methods 1 and 2 were 24.9 dB and 19.5 dB, respectively. These results clearly indicate that Method 3 was the most effective processing technique in this study. Actually, with a suitable compounding sequence in cooperation with proper processing conditions, as well as a suitable iPP/TPU ratio and CNT content, lightweight and high-performance EMI shielding polymer nanocomposites could be manufactured; such materials have promising applications in some related areas.

## 4. Conclusions

In summary, we have developed an easy and effective strategy for the design of a two-phase system and selective localization of MWCNT in a bicontinuous phase structure and thus successfully prepared conductive iPP/TPU/MWCNT@ composites with a low percolation threshold by melt blending. When the mass ratio of iPP/TPU was 4:6 and the content of the compatibilizer PP-g-MAH was 1 wt.%, with the sequential processing method, the composites had the most complete bicontinuous phase structure and achieved high electrical conductivity and electromagnetic shielding properties at low filler incorporation levels. The EMI shielding value reached 37.8 dB for samples with a thickness of 2.5 mm at a tested frequency of 26.5 GHz. This also confirms the conjecture that the construction of the bicontinuous phase structure helps to improve the electrical conductivity and electromagnetic shielding properties of composites.

## Data Availability

Data will be made available on request.
